# Effects of carbon-based additives and ventilation rate on nitrogen loss and microbial community during chicken manure composting

**DOI:** 10.1371/journal.pone.0229880

**Published:** 2020-09-23

**Authors:** Ruixue Chang, Yanming Li, Qing Chen, Xiaoyan Gong, Zicheng Qi

**Affiliations:** 1 College of Resources and Environmental Sciences, China Agricultural University, Beijing, China; 2 Shandong Academy of Agricultural Machinery Sciences, Jinan, Shandong Province, China; Gifu University, JAPAN

## Abstract

Aerobic composting is a sustainable method for chicken manure recycling, while its unsuitable porosity and carbon to nitrogen ratio (C/N) may result in high nitrogen loss and incomplete composting. With the aim to investigate the effects of carbon-based additives and two ventilation rates on chicken manure composting and microbial community, two series of treatments were set up for chicken manure composting, in order to investigate their effects on the biodegradation process, ammonia (NH_3_) emission, nitrogen loss, physiochemical properties and microbial community. The results showed that additives and ventilation rates set in the current study influenced the carbon dioxide (CO_2_) production from the 2^nd^ week and also the physiochemical parameters during the entire process, while no inhibitory effect on the maturity were observed. With woody peat as additive, the NH_3_ emission amount and nitrogen loss rate were shown as 15.86 mg and 4.02%, less than those in other treatments, 31.08–80.13 mg and 24.26–34.24%, respectively. The high aeration rate increased the NH_3_ emission and nitrogen loss, which were varied when the additives were different. The terminal restriction fragment length polymorphism (T-RFLP) results showed that the additives and the ventilation rates changed the microbial community, while the prominent microbial clones belonged to the class of *Bacilli* and *Clostridia* (in the phylum of *Firmicutes*), and *Alphaproteobacteria*, *Deltaproteobacteria* and *Gammaproteobacteria* (in the phylum of *Proteobacteria*). *Bacillus spp*. was observed to be the most dominant bacteria in all the composting stages and treatments. It was concluded that woody peat could improve chicken manure composting more than other additives, especially on reducing nitrogen loss, meanwhile 0.18 L‧min^-1^‧kg^-1^ DM was suitable for various additives. Therefore, suitable additive and aeration rate could be used in practical application, which could significantly reduce nitrogen loss without influence on the compos maturity process.

## 1. Introduction

With the rapid development of intensive and large-scale chicken farms in China, nearly 102 million tons of chicken manure (dry weight) were produced in 2016 [1], which has become one of the main causes of non-point source pollution threatening the environment (i.e. emissions to water and air) [2]. Aerobic composting shows potential for the conversion of the chicken manure into biofertilizers and reduce the potential pollution [3, 4]. Whereas the characteristics of poor porosity and low C/N ratio, limit the oxygen consumption and organic matter (OM) degradation, resulting in high nitrogen loss during chicken manure composting [5]. However, the high temperature (>45°C) reached during the composting process may help to shift the NH_4_ to NH_3_ [6, 7] and inhibit nitrification at the same time, thereby increasing NH_3_ volatilization [8]. Therefore, it is always of importance to improve the efficiency and product quality of chicken manure composting, by improving the selected materials and technique parameters.

Various composting studies have investigated emission (N_2_O and NH_3_) abatement induced by additives such as, zeolite [9, 10], bentonite [11], medical stone [12], woody peat and biochar [7, 13, 14], saw dust [15], pine bark [16] and peanut hull [17] were studied in composting experiments of various feedstock materials to improve the porosity and C/N ratio, so that the emission of N_2_O and NH_3_ could be reduced [14, 18]. Most of these studies gave detailed descriptions of the composting process, by analyzing temperature, gaseous emissions, microbial community dynamics, elements (C, N, P) and humic substance transformation, with the aim to find suitable composting additives. Few studies have compared the effect of varied biodegradable OM content in these additives, which may have a significantly positive impact on the composting process and the temperature [19, 20].

Meanwhile a suitable aeration rate in composting could provide sufficient oxygen, regulate reactor temperature, control water loss and reduce odorous matter emissions [21–23]. Suitable aeration rates vary in different studies with different feedstocks, reactors or additives [24–26]. Especially since different additives used in the materials may significantly influence the temperature and OM degradation, so that a great impact on nitrogen loss would be observed [7]. Based on this, the aeration rate would change the influence of additives on organic degradation, temperature variation and also on nitrogen loss.

In the present study, 2 series of 30-d chicken manure composting were carried out in a lab-scale composting system, with the aim to (1) explore the effects of carbon-based additives with different biodegradable OM, (2) investigate the effects of ventilation rates with the same or different additives on OM degradation, composting process and the microbial community changes.

## 2 Material and methods

### 2.1 Set-up of experiment

The feedstock (chicken manure) and additives (corn straw, saw dust, pine bark and peanut hull) used in the current study were collected from a greenhouse or farmland in Beijing, China. Powder woody peat was supplied by View Sino international Ltd. Prior to the composting process, the additives were air dried and cut into 2–3 cm to enable good mixing of raw materials. Main characteristics of the raw materials are shown in Table 1.

**Table 1:** Physical and chemical properties of the materials.

Materials	Total carbon content / %	Total nitrogen content / %	C/N	Moisture / %	Cellulose / %	Lignin / %
Chicken manure	37.63	4.33	8.69	81.28	13.06	9.06
Wheat straw	62.35	0.73	85.41	7.14	42.03	8.86
Woody peat	62.84	0.59	196.51	14.04	2.92	28.91
Saw dust	67.70	0.41	165.12	8.33	50.85	17.39
Pine bark	73.52	0.44	167.09	8.79	61.62	31.22
Peanut hull	52.77	1.06	49.78	7.85	64.10	25.65

**Fig 1:**
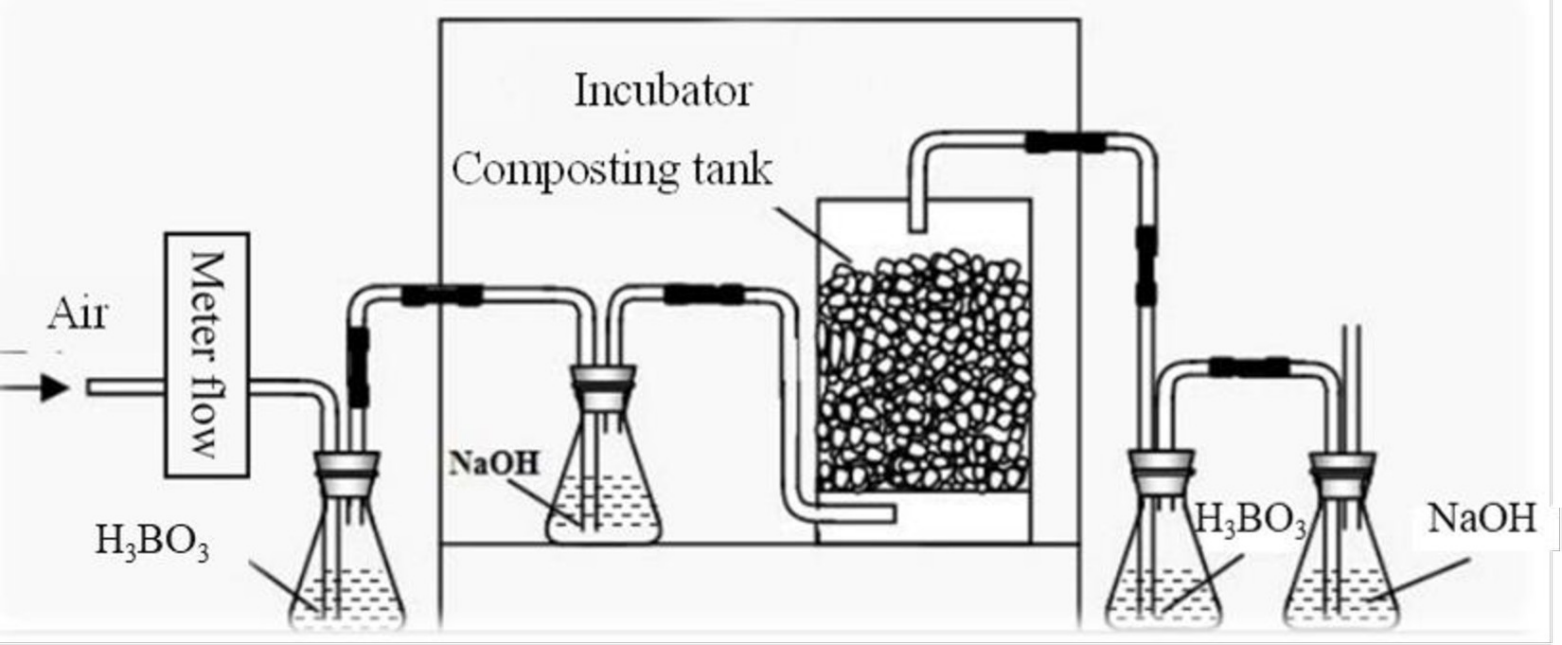
Diagram of the composting system.

The experiments were conducted in the lab of China Agricultural University, Beijing, China. The bench-scale compost system (Fig 1) used in this study was designed to simulate the temperature (50°C), moisture (60%wet weight basis) and forced ventilation of the composting process, which could be vulnerable to external effects (e.g. heat loss) [19]. The reactor capacity is 5L, connected with solutions of NaOH and H_3_PO_4_ before and after each reactor used to absorb the NH_3_ and CO_2_ in the air before and after the composting. There were two series of treatments sets in the current experiments (shown in Table 2), with different carbon additives and different aeration rates, respectively.

**Table 2:** Experiment design of different resource carbon.

Treatments		Raw materials mixed ratio (Fresh matter)	C/N	Ventilation rate / L‧min^-1^‧kg^-1^ DM
Series 1	T1	Chicken manure: wheat straw = 1:0.323	25:1	0.18
	T2	Chicken manure: woody peat = 1:0.321		
	T3	Chicken manure: saw dust = 1:0.251		
	T4	Chicken manure: pine bark = 1:0.232		
	T5	Chicken manure: peanut hull = 1:0.548		
Series 2	T1	Chicken manure: wheat straw = 1:0.323	25:1	0.18
	S1			0.36
	T3	Chicken manure: saw dust = 1:0.251		0.18
	S3			0.36
	T5	Chicken manure: peanut hull = 1:0.548		0.18
	S5			0.36

### 2.2 Samples collection and analysis

During the composting, CO_2_ produced during composting was trapped by bubbling the exit gas through a solution of NaOH; the amount of CO_2_ in the traps was determined by titration with standard H_2_SO_4_ [27].

The solid samples were collected on the day 0, 3, 7, 14, 21, 28 and 35 after being mixing well. Each sample was thoroughly mixed and then divided into two parts: one part was air-dried to analyze physicochemical characteristics, like total nitrogen (TN) and ash content; the other part was stored in the freezer at -20°C for the determination of other parameters, like pH value, Electric Conductivity (EC), Germination Index (GI), extractable ammonium and microbial community. Measurement of these parameters and calculation methods were shown in previous studies [19].

A 1:5 aqueous extract (w/v) of the fresh composts with 2N KCl solution was used for the analysis of extractable ammonium (NH_4_^+^-N), and NH_4_^+^-N was analyzed by SEAL Analytical (BL-TECH).

### 2.3 Analysis of microbial community

Total community DNA was extracted from 0.5 g compost samples using the FastPrep DNA kit (MP Biomedicals, Santa Ana, CA) according to the manufacturer’s protocol [28]. The extracted DNA solutions were diluted with suitable time. The 16S rRNA genes were amplified using universal bacterial primers: 27f forward (5’-AGAGTTTGATCCTGGCTCAG-3’) and 907r (5’-CCGTCAATTCMTTTGAGTT -3’) reverse. The 27f forward primer was labeled with 6-carboxyfluorescein (FAM). Each PCR reaction mixture contained 50 μl liquid: 37.5 μl dd H_2_O, 10* PCR reaction buffer 5μl (Tiangen Biotech, Beijing), dTNPs 4μl, 27f-FAM 0.75μl, 907r 1.5 μl, BSA 0.5μl, rTaq DNA polymerase0.5 μl (TakaRa), DNA template 2μl. The reaction mixture was incubated at 94°C for 4 min, and then cycled 30 times through three steps: denaturing (94°C; 45 s), annealing (52°C; 45 s), and primer extension (72°C; 60 s) in a PTC-100 thermal cycler. Then the last step was 10 mins primer extension. Amplification product sizes were verified by electrophoresis in 2.0% agarose and ethidium bromide staining. To obtain sufficient DNA for T-RFLP analysis and to minimize PCR bias, amplicons from three PCR runs for each root sample were combined [29] and then purified using a PCR purification kit (PCR Clean-up Kit; PROMEGA Inc., Wisconsin, USA).

To construct bacterial 16S rRNA gene-based clone libraries, DNA samples extracted from five compost samples with the richest bacteria diversity from different additive treatments were prepared respectively. The PCR amplification used the same primers as those indicated above. PCR products were purified and ligated into the pMD19-T Vector (TakaRa) according to the manufacturer’s instructions. A 1 ml suction head was used to blow and absorb the bacteria at the bottom of the centrifuge, and then 20–40 μl of the cells were coated on LB AGAR plate medium containing X-Gal, IPTG and Amp for an overnight culture at 37°C, to form a single colony. White clones were selected and underlined on LB-Amp plates and cultured overnight at 37°C. The screened positive clones were sent to the sequencing company for sequencing, which were screened with the primers M13-47 (5’-CAGCAC TGA CCC TTT TGG GAC CGC-3’) and RV-M (5’GAG CGG ATA ACA ATT TCA CAC AGG-3’). Put the results into NCBI GeneBank database and performed Blast search to obtain similar gene sequences. A Phylogenetic Tree was constructed with MEGA software and NJ method (neighbor-joining).

### 2.4 Statistical analysis

All the results were summarized and figured in Excel. Statistical comparisons were performed using SPSS v.18.0 software with the two-way ANOVA analysis of variance test. Probability was defined with a least significant difference at two sides of P < 0.05.

## 3. Results and discussion

### 3.1 OM biodegradation and CO_2_ production

**Fig 2:**
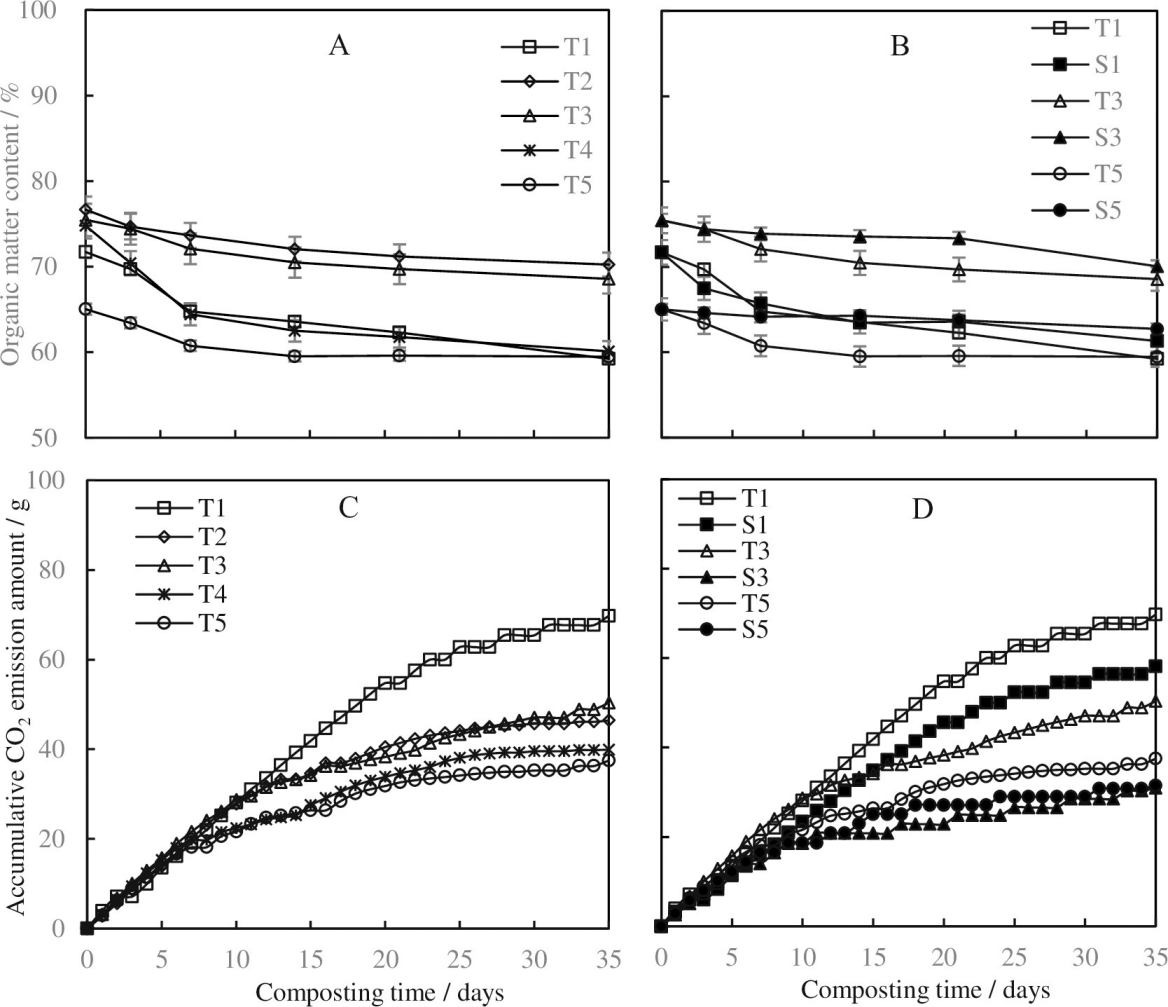
Effects of carbon-based additives (A, C) and ventilation rate (B, D) on organic matter degradation and CO_2_ emissions during chicken manure composting.

Rapid decrease of OM and increase of cumulative CO_2_ amount coincided with the variation of temperature during composting [30]. As shown in Fig 2A, additives used in the current study changed the OM contents in the initial materials, while all of them decreased along with the composting process. At the end of the process, the contents in T1, T4 and T5 were almost the same, while T2 and T3 were similar. The aeration rate of 0.36 L‧min^-1^‧kg^-1^ DM decreased the OM degradation during the composting process (Fig 2B) in all the composting piles. Similar CO_2_ emission trends were observed in T1-T5 in the first 7 days, suggesting the easily-degraded OM was quickly degraded and transferred to CO_2_ (Fig 2C). Additives used in T1-T5 changed the CO_2_ emission from Day 7. Less CO_2_ emission amounts were observed when the ventilation rate was 0.36 L‧min^-1^‧kg^-1^ DM in S1, S3 and S5 (Fig 2D), suggested that the 0.18 L‧min^-1^‧kg^-1^ was more suitable for the composting process in the current study. Based on the changes of OM and CO_2_ emission, the easily-degraded OM in T2-T5 were less than that in T1. The concentration of cellulose and lignin was higher in saw dust, pine bark and peanut hull, when compared with wheat straw, while cellulose and lignin were hard to be biodegraded directly. Woody peat is rich in carbon and humus, but they were unavailable for microbial biodegradation, so that the CO_2_ amount was lower than that in T1. Carbon additives, like biochar or woody peat, had shown similar results in vegetable wastes or sewage sludge composting [14].

In a previous study, the low aeration rate (0.3 L‧min^-1^‧kg^-1^ DM) corresponded to a higher and longer thermophilic phase than the high aeration rate (0.9 L‧min^-1^‧kg^-1^ DM) in the ventilation range of 0.3–0.9 L‧min^-1^‧kg^-1^ DM [31]. Some other studies recommended various aeration methods and rates for different raw materials, like 0.5 L‧min^-1^‧kg^-1^ DM in the composting of chicken manure and sawdust [32], 0.25 L‧min^-1^‧kg^-1^ DM in the composting of dairy manure with rice straw [33], 0.43–0.86 L‧min^-1^‧kg^-1^ DM in the composting of food waste [34], etc. These suggested the aeration rate in composting should be set according to the compost material and composting process, based on the oxygen needed and supplied during the process.

### 3.2 Physiochemical characteristics

**Fig 3:**
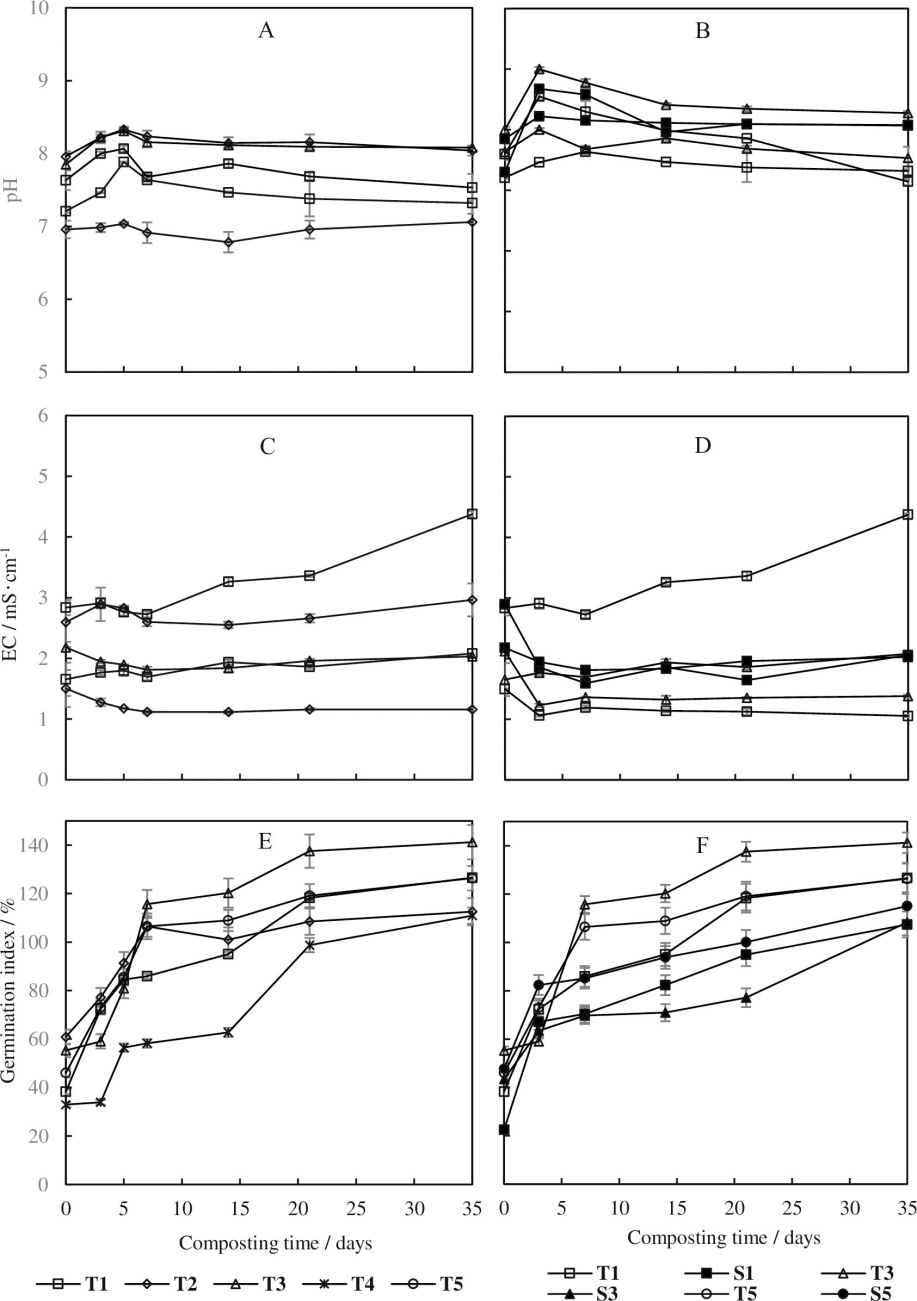
Effects of carbon-based additives (A, C, E) and ventilation rate (B, D, F) on physiochemical characteristics during chicken manure composting.

The appropriate pH range for maintaining high microbial activity during composting is 7–8, which would be changed along with the biodegradation of OM. The complex components were degraded to organic acids and then to CO_2_, meanwhile CO_2_, NH_3_, other gases and volatile organic acids were emitted from the composting system [35]. As shown in Fig 3A, the pH values in all the treatments were in the range of 6.8~8.4, suggesting the carbon additives changed the pH value and biodegradation process. The final values of the products were all in the range of 7.0~8.2, which were good for agricultural use [36]. The pH value in T2 was lower than others, indicating the potential advantage of woody peat, to reduce the NH_3_ emission by decreasing the material pH value. An increase of the aeration rate quickly increased the pH values (Fig 3B), as the gases and volatile organic acids were forced to be emitted more frequently than in the low aeration rate treatments. The final pH values of products in S1-S3 were higher than those in T1-T3.

A slight decrease was shown in the first several days for all treatments, followed by a stable value until the end (Fig 3C and 3D). The carbon additives influenced the EC variation, while they were always under 4 mS·cm^-1^, except 4.37 mS·cm^-1^ in T1. The results reflected that the products could be used as organic amendments or organic fertilizer in soil [37], as they have no inhibitory effects on plant growth. Woody peat used in T2 reduced the EC in the whole process, because of the absorption caused by its rich humic acid. The rapid emission of gases and volatile organic acids in treatments with high aeration rate also reduced the EC of products.

To avoid the toxic effects on plant growth resulting from toxic substances, such as short-chain fatty acids, GI is always used as an important index to evaluate whether compost is mature enough. A minimum value of 80% is considered to indicate the compost maturity is at an extraction ratio of 1:5 (compost: water wet w/v). As shown in Fig 3E and 3F, the GI values increased with the decomposition of toxic materials, especially in the first 7 days. Nearly all the GI values were higher than 80% in the treatments, except T4 in series Ⅰ, and S1 and S3 in series Ⅱ. Then the GI values keep increasing slightly until the end of the process, with the GI values over 100%. The results indicated that the five additives chosen in the current study could adjust the free air space and C/N of chicken manure to improve the composting process. The higher aeration rate (0.36 L‧min^-1^‧kg^-1^ DM) decreased the maturity process. However, it was opposite in a pig manure composting, in which the suitable aeration was 0.48 L‧min^-1^‧kg^-1^ DM even the lower aeration rate (0.24 L‧min^-1^‧kg^-1^ DM) had better performance on biodegradation [38]. The reason may be complex: one is that higher aeration rate could supply enough O_2_ than a lower one when the aeration is intermittent; the other is that lower C/N of the mixed materials (< 20) causes high concentration of TAN (total ammonium nitrogen) in the materials, which would inhibit the seedling.

### 3.3 NH_3_ emission and nitrogen concentrations

**Fig 4:**
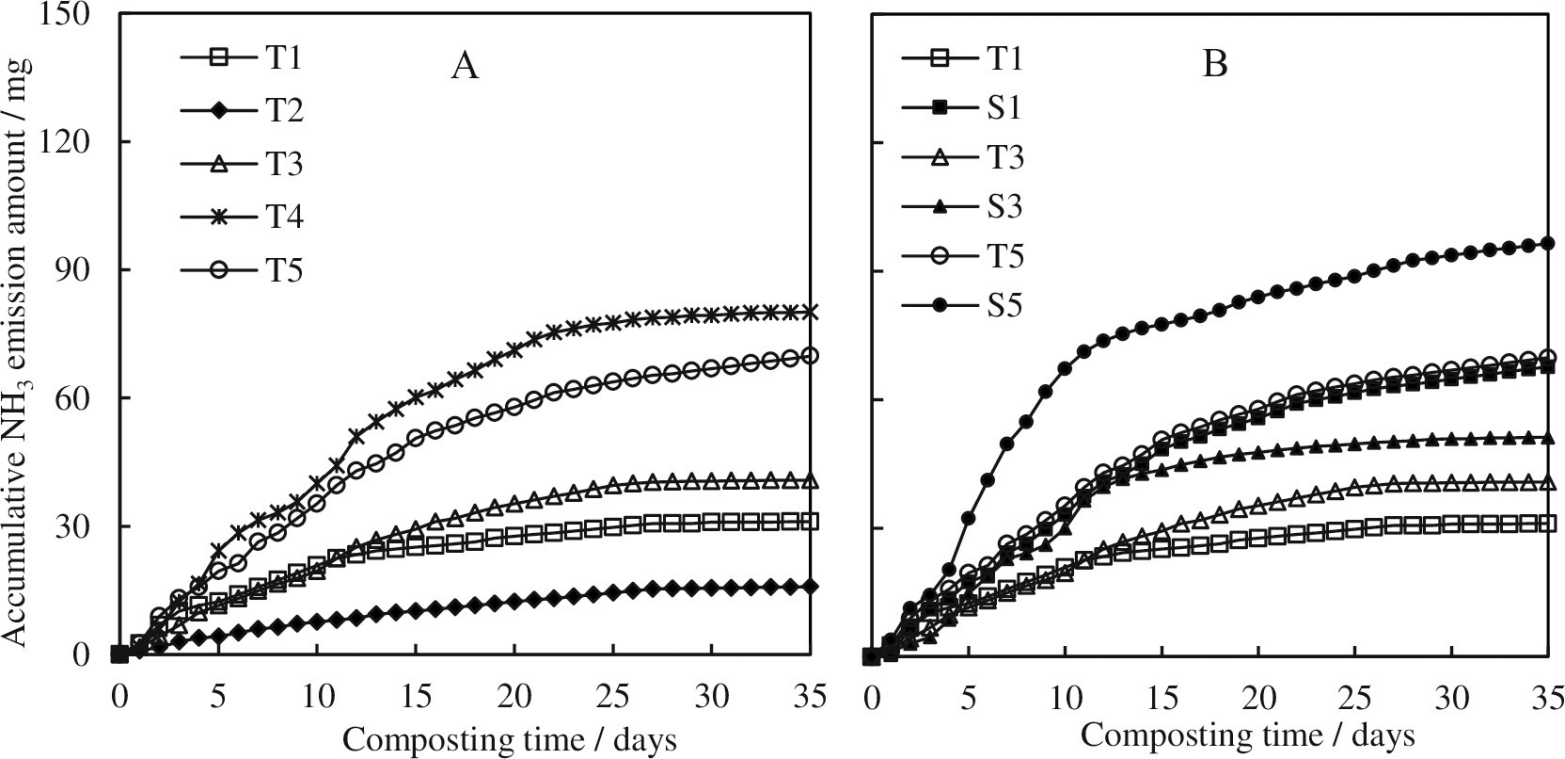
Effects of carbon-based additives (A) and ventilation rate (B) on NH_3_ emissions during chicken manure composting.

The changes of accumulative NH_3_ emission amount during the composting in the two series were shown in Fig 4. In all the treatments, the accumulative amounts rapidly increased and then the increasing rates slowed down. Additives used in series Ⅰ changed the emission rate and the lasting time, as shown in Fig 4A. Significantly lower cumulative NH_3_ emission in T2 (15.86 mg) was observed than those in other treatments. This should be related to the characteristics of woody peat, low pH and rich in humic acid, which contributed to the absorption of NH_4_^+^. More cumulative NH_3_ amounts were observed in T3-T5 than that in T1, which may be related to the lower biodegradable organic carbon in the mixed materials. There are high concentrations of lignocellulose in saw dust, pine bark and peanut hull, which may decrease the biodegradable C/N and increase the NH_3_ emission. When it came to series Ⅱ, S1-S3 had significantly higher cumulative NH_3_ losses than T1-T3, respectively, suggesting the increased aeration rate in the current study would lead to more NH_3_ emission.

**Fig 5:**
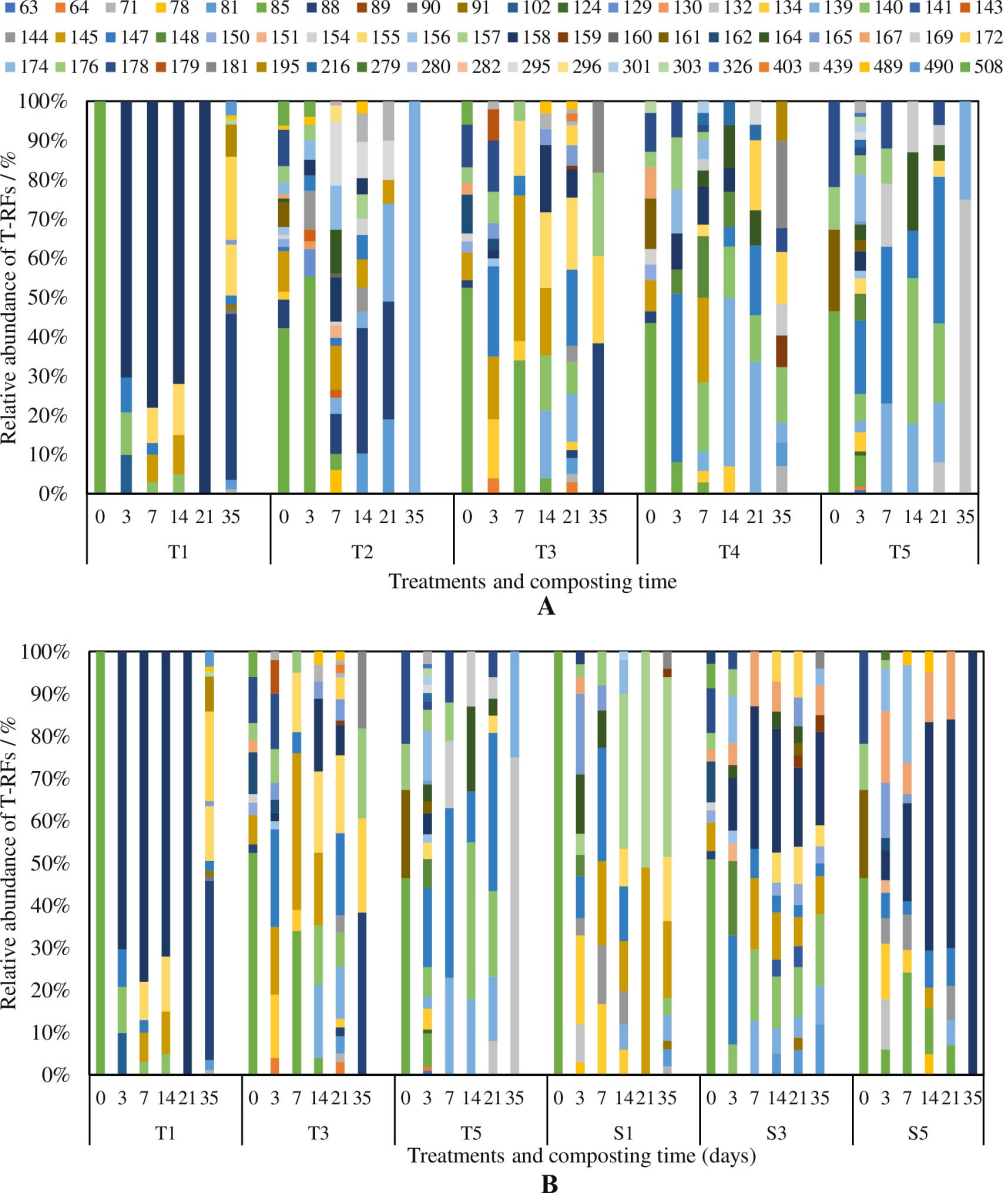
Effects of carbon-based additives (A) and ventilation rate (B) on relative abundance of T-RFs during chicken manure composting.

TN concentrations of initial materials and products were shown in Table 3. Except T5 and S5, the TN concentrations were higher in the products of all treatments, because of the concentration effect caused by the significant organic decomposition [10]. Peanut hull, with high concentrations of cellulose and lignin, was used as carbon additive in T5 and S5, in which the NH_3_ emission were higher than in other treatments, shown as 69.79 mg and 96.41 mg (Table 3). Meanwhile, less organic decomposition decreased the concentration effect of the mixed material. There are several forms of nitrogen in the compost, among which organic nitrogen (Nor) and nitrate nitrogen (NO_3_^—^N) are normally stable in the materials and called stable nitrogen, while NH_4_^+^-N may transform to NO_3_^—^N or be emitted as NH_3_, when the temperature was decreased or during the utilization of the compost in arable land. High NH_4_^+^-N concentration in the materials always contributes to a high NH_3_ emission rate, which was consistent in our study except for T2. The different results indicated the important function of woody peat to absorb and fix the NH_4_^+^-N, because of its porous structure and richness of humus acid. Similar results could be observed when biochar was used during composting [39]. More NH_4_^+^-N in treatments T3 and T5 than S3 and S5 (shown in Table 3), suggested that the high aeration rate helped to transfer NH_4_^+^-N into NH_3_ ventilation, so that more NH_3_ ventilation was observed in Fig 5 and Table 2. Meanwhile the total nitrogen loss rates were higher in treatments with high aeration rate resulting from high NH_3_ ventilation.

**Table 3:** Nitrogen transformation and nitrogen loss in all treatments.

	Treatments	TN / g·kg^-1^		Ammonium nitrogen / mg·kg^-1^		Total NH_3_ emission amount / mg	TN loss / %
		Initial	End	Initial	End		
Series 1	T1	21.55	23.52	759.07	105.40	31.08	24.26
	T2	19.03	21.60	1310.33	840.73	15.86	4.02
	T3	23.89	26.44	963.93	212.07	40.79	28.45
	T4	19.85	20.65	1632.33	817.20	80.13	34.24
	T5	20.88	16.68	1236.73	877.80	69.79	32.74
Series 2	T1	21.55	23.52	759.07	105.40	31.08	24.26
	S1	21.55	22.19	759.07	138.80	67.66	35.75
	T3	23.89	26.44	963.93	212.07	40.79	28.45
	S3	23.68	25.06	963.93	154.80	51.21	29.51
	T5	20.88	16.68	1236.73	877.80	69.79	32.74
	S5	20.88	16.73	1236.73	494.30	96.41	36.24

### 3.4 Structure of microbial community

Different additives used in the current study changed the biodiversity during the composting process (Fig 5A). At the beginning, the most abundant T-RF was 85bp, with the ratio of 100%, 56%, 53%, 44% and 47%, in T1-T5 respectively. In all the 60 T-RFs shown in the figures, 139bp, 145bp, 147bp, 174bp, 176bp and 178bp had high relative abundances in two or more treatments in different stages, indicated these T-RFs should be related with the OM biodegradation. Meanwhile, 158bp (in T1), 295bp (in T2), 155bp (in T3), 140bp (in T4) and 132bp (in T5) had high relative abundance in specific treatment, which was related to this additive only. The increase of the ventilation rate contributed to higher biodiversity in all the treatments (Fig 5B). The results suggested the additives and aeration rates in the current study influenced the microbial population, resulting in a different biodegradation process.

**Fig 6:**
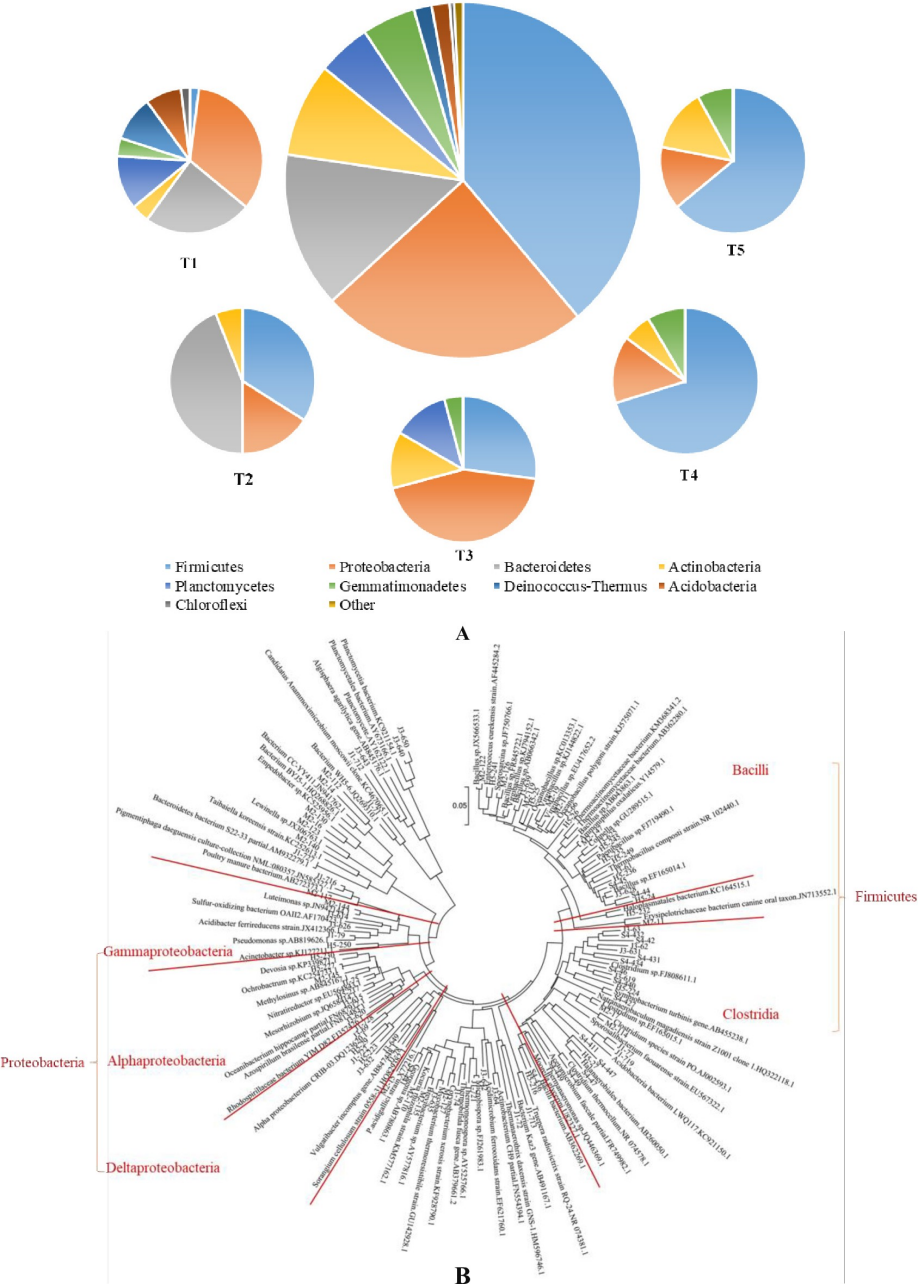
Composition of 247 sequences in 5 compost samples based on the bacteria clone library analysis (A) and their phylogenetic analysis (shown as OUT and restriction enzyme cutting site) (B).

Constructing a bacterial clone library could help to understand the species distribution in the composting system. Five of the compost samples in different treatments were selected to construct the clone library, in which *Firmicutes*, *Proteobacteria*, *Bacteroides* and *Actinomycetes* accounted for 85.83% in the total 247 sequences (Fig 6A). *Bacillus spp*., belonging to the phylum of *Firmicutes*, was primary in all the sequences, because of its characteristics of high-temperature resistance and OM biodegradation ability [40]. The result of the phylogenetic analysis affiliated with uncultured groups using the neighbor-joining method is shown in Fig 6B. Carbon additives used in different treatments influenced the clones, similar as shown in Fig 6A. Prominent clones belonged to an uncultured group in the phylum *Firmicutes* (class of *Bacilli* and *Clostridia*) and *Proteobacteria* (class of *Alphaproteobacteria*, *Deltaproteobacteria* and *Gammaproteobacteria*), which were also observed in mature compost samples in previous studies [39–41]. High proportions of *Actinobacteria* in the samples of T1 and T3, suggested that the addition of corn straw or saw dust may facilitate the growth of *Actinobacteria* and accelerate the degradation of lignocelluloses during the maturity stage [39].

Based the results above, it can be concluded that the additives and aeration rates used in the current study changed the microbial community, which may be one of the main reasons for the change of biodegradation and the emission amounts of CO_2_ and NH_3_. However, the composting process and the main functional microbes remained similar in different treatments even they were influenced by the additives and aeration rates.

## 4. Conclusion

Composting using any of the additives in the current study improved the biodegradation process and influenced the pH, EC and GI values of the product. Among them, woody peat had the best effect on reducing NH_3_ emission and nitrogen loss. The aeration rate of 0.18 L‧min^-1^‧kg^-1^ DM was more suitable than 0.36 L‧min^-1^‧kg^-1^ DM for chicken manure composting, which was also better for controlling NH_3_ emission and nitrogen loss. The prominent clones of the compost samples belonged to the phylum Firmicutes (class of *Bacilli* and *Clostridia*) and Proteobacteria (class of *Alphaproteobacteria*, *Deltaproteobacteria* and *Gammaproteobacteria*). Among them, Bacillus spp. was always the most important one, even carbon-based additive and ventilation rate all made influences on the microbial community. Therefore, woody peat could be used as additive instead of corn straw, and suitable ventilation rates could reduce the NH_3_ emission and nitrogen loss.

## References

[1] Jia W, Qin W, Zhang Q, Wang X, Ma Y, Chen Q. Evaluation of crop residues and manure production and their geographical distribution in China. J Clean Prod. 2018;188:954–65.

[2] Shi M, Wei Z, Wang L, Wu J, Zhang D, Wei D, et al Response of humic acid formation to elevated nitrate during chicken manure composting. Bioresour Technol. 2018;258:390–4. 10.1016/j.biortech.2018.03.056 29571890

[3] Hagemann N, Subdiaga E, Orsetti S, de la Rosa JM, Knicker H, Schmidt HP, et al Effect of biochar amendment on compost organic matter composition following aerobic composting of manure. Sci Total Environ. 2018;613–614:20–9. 10.1016/j.scitotenv.2017.08.161 28892724

[4] Wu J, Zhao Y, Wang F, Zhao X, Dang Q, Tong T, et al Identifying the action ways of function materials in catalyzing organic waste transformation into humus during chicken manure composting. Bioresour Technol. 2020;303:122927 10.1016/j.biortech.2020.122927 32050125

[5] Wang K, Li W, Li X, Ren N. Spatial nitrifications of microbial processes during composting of swine, cow and chicken manure. Sci Rep. 2015;5:14932 10.1038/srep14932 26442637 PMC4595641

[6] Pagans E, Barrena R, Font X, Sanchez A. Ammonia emissions from the composting of different organic wastes. Dependency on process temperature. Chemosphere. 2006;62(9):1534–42. 10.1016/j.chemosphere.2005.06.044 16085275

[7] Chang R, Li Y, Chen Q, Guo Q, Jia J. Comparing the effects of three in situ methods on nitrogen loss control, temperature dynamics and maturity during composting of agricultural wastes with a stage of temperatures over 70 degrees C. J Environ Manage. 2019;230:119–27. 10.1016/j.jenvman.2018.09.076 30278275

[8] Nigussie A, Bruun S, Kuyper TW, de Neergaard A. Delayed addition of nitrogen-rich substrates during composting of municipal waste: Effects on nitrogen loss, greenhouse gas emissions and compost stability. Chemosphere. 2017;166:352–62. 10.1016/j.chemosphere.2016.09.123 27710881

[9] Awasthi MK, Wang Q, Ren X, Zhao J, Huang H, Awasthi SK, et al Role of biochar amendment in mitigation of nitrogen loss and greenhouse gas emission during sewage sludge composting. Bioresour Technol. 2016;219:270–80. 10.1016/j.biortech.2016.07.128 27497088

[10] Chan MT, Selvam A, Wong JW. Reducing nitrogen loss and salinity during 'struvite' food waste composting by zeolite amendment. Bioresour Technol. 2016;200:838–44. 10.1016/j.biortech.2015.10.093 26590758

[11] Wang Q, Wang Z, Awasthi MK, Jiang Y, Li R, Ren X, et al Evaluation of medical stone amendment for the reduction of nitrogen loss and bioavailability of heavy metals during pig manure composting. Bioresour Technol. 2016;220:297–304. 10.1016/j.biortech.2016.08.081 27589824

[12] Wang Q, Awasthi MK, Zhao J, Ren X, Li R, Wang Z, et al Improvement of pig manure compost lignocellulose degradation, organic matter humification and compost quality with medical stone. Bioresour Technol. 2017;243:771–7. 10.1016/j.biortech.2017.07.021 28711806

[13] Zhang J, Lu F, Shao L, He P. The use of biochar-amended composting to improve the humification and degradation of sewage sludge. Bioresour Technol. 2014;168:252–8. 10.1016/j.biortech.2014.02.080 24656550

[14] Awasthi MK, Wang M, Chen H, Wang Q, Zhao J, Ren X, et al Heterogeneity of biochar amendment to improve the carbon and nitrogen sequestration through reduce the greenhouse gases emissions during sewage sludge composting. Bioresour Technol. 2017;224:428–38. 10.1016/j.biortech.2016.11.014 27843087

[15] Sharma D, Yadav KD, Kumar S. Role of sawdust and cow dung on compost maturity during rotary drum composting of flower waste. Bioresour Technol. 2018;264:285–9. 10.1016/j.biortech.2018.05.091 29852418

[16] Brito LM, Mourão I, Coutinho J, Smith SR. Co-composting of invasive Acacia longifolia with pine bark for horticultural use. Environ Technol. 2015;36(13):1632–42. 25559143 10.1080/09593330.2014.1002863

[17] Erickson MC, Liao J, Boyhan G, Smith C, Ma L, Jiang X, et al Fate of manure-borne pathogen surrogates in static composting piles of chicken litter and peanut hulls. Bioresour Technol. 2010;101(3):1014–20. 10.1016/j.biortech.2009.08.105 19783430

[18] Mao H, Lv Z, Sun H, Li R, Zhai B, Wang Z, et al Improvement of biochar and bacterial powder addition on gaseous emission and bacterial community in pig manure compost. Bioresour Technol. 2018;258:195–202. 10.1016/j.biortech.2018.02.082 29525594

[19] Chang R, Guo Q, Chen Q, Bernal MP, Wang Q, Li Y. Effect of initial material bulk density and easily-degraded organic matter content on temperature changes during composting of cucumber stalk. J Environ Sci (China). 2019;80:306–15. 10.1016/j.jes.2017.10.004 30952348

[20] Wu D, Wei Z, Qu F, Mohamed TA, Zhu L, Zhao Y, et al Effect of Fenton pretreatment combined with bacteria inoculation on humic substances formation during lignocellulosic biomass composting derived from rice straw. Bioresour Technol. 2020;303:122849 10.1016/j.biortech.2020.122849 32035389

[21] Alkoaik FN. Integrating aeration and rotation processes to accelerate composting of agricultural residues. PLoS One. 2019;14(7):e0220343 10.1371/journal.pone.0220343 31344136 PMC6657913

[22] Ge M, Zhou H, Shen Y, Meng H, Li R, Zhou J, et al Effect of aeration rates on enzymatic activity and bacterial community succession during cattle manure composting. Bioresour Technol. 2020;304:122928 10.1016/j.biortech.2020.122928 32106020

[23] Zhang H, Li G, Gu J, Wang G, Li Y, Zhang D. Influence of aeration on volatile sulfur compounds (VSCs) and NH3 emissions during aerobic composting of kitchen waste. Waste Manage. 2016;58:369–75. 10.1016/j.wasman.2016.08.022 27595496

[24] Xu Z, Zhang LL, Li J. Effect of Different Aeration Rates on the Composting Process and Its Enzymatic Activities. J Residuals Sci Technol. 2012;9(3):107–12.

[25] Shen Y, Ren L, Li G, Chen T, Guo R. Influence of aeration on CH4, N2O and NH3 emissions during aerobic composting of a chicken manure and high C/N waste mixture. Waste Manag. 2011;31(1):33–8. 10.1016/j.wasman.2010.08.019 20888749

[26] Jiang T, Li G, Tang Q, Ma X, Wang G, Schuchardt F. Effects of aeration method and aeration rate on greenhouse gas emissions during composting of pig feces in pilot scale. J Environ Sci (China). 2015;31:124–32. 25968266 10.1016/j.jes.2014.12.005

[27] Michel FC, Reddy CA. Effect of Oxygenation Level on Yard Trimmings Composting Rate, Odor Production, And Compost Quality In Bench-Scale Reactors. Compost Sci Util. 1998;6(4):6–14.

[28] Feng Y, Xu Y, Yu Y, Xie Z, Lin X. Mechanisms of biochar decreasing methane emission from Chinese paddy soils. Soil Biol Biochem. 2012;46:80–8.

[29] Clément K, Vaisse C, Lahlou N, Cabrol S, Pelloux V, Cassuto D, et al A mutation in the human leptin receptor gene causes obesity and pituitary dysfunction. Nature. 1998;392(6674):398–401. 10.1038/32911 9537324

[30] Wong JWC, Mak KF, Chan NW, Lam A, Liao XD. Co-composting of soybean residues and leaves in Hong Kong. Bioresour Technol. 2001;76(2):99–106. 10.1016/s0960-8524(00)00103-6 11131806

[31] Qasim W, Moon BE, Okyere FG, Khan F, Nafees M, Kim HT. Influence of aeration rate and reactor shape on the composting of poultry manure and sawdust. J Air Waste Manag Assoc. 2019;69(5):633–45. 10.1080/10962247.2019.1569570 30640581

[32] Gao M, Bing L, An Y, Liang F, Yang L, Sun Y. The effect of aeration rate on forced-aeration composting of chicken manure and sawdust. Bioresour Technol. 2010;101(6):1899–903. 10.1016/j.biortech.2009.10.027 19897360

[33] Li X, Zhang R, Pang Y. Characteristics of dairy manure composting with rice straw. Bioresour Technol. 2008;99(2):359–67. 10.1016/j.biortech.2006.12.009 17320381

[34] Lu S.G., Imai T., Ukita M. Effect of enforced aeration on in-vessel food waste composting. Environ Technol. 2001;22(10):1177–82. 10.1080/09593332208618200 11766039

[35] Eklind Y, Kirchmann H. Composting and storage of organic household waste with different litter amendments. II: nitrogen turnover and losses. Bioresour Technol. 2000;74(2):125–33.

[36] Aulinas Masó M, Bonmatí Blasi A. Evaluation of composting as a strategy for managing organic wastes from a municipal market in Nicaragua. Bioresour Technol. 2008;99(11):5120–4. 10.1016/j.biortech.2007.09.083 18006301

[37] Liu D, Zhang R, Wu H, Xu D, Tang Z, Yu G, et al Changes in biochemical and microbiological parameters during the period of rapid composting of dairy manure with rice chaff. Bioresour Technol. 2011;102(19):9040–9. 10.1016/j.biortech.2011.07.052 21835612

[38] Guo R, Li G, Jiang T, Schuchardt F, Chen T, Zhao Y, et al Effect of aeration rate, C/N ratio and moisture content on the stability and maturity of compost. Bioresour Technol. 2012;112:171–8. 10.1016/j.biortech.2012.02.099 22437050

[39] Qiu X, Zhou G, Zhang J, Wang W. Microbial community responses to biochar addition when a green waste and manure mix are composted: A molecular ecological network analysis. Bioresour Technol. 2019;273:666–71. 10.1016/j.biortech.2018.12.001 30528727

[40] Koyama M, Nagao N, Syukri F, Rahim AA, Kamarudin MS, Toda T, et al Effect of temperature on thermophilic composting of aquaculture sludge: NH3 recovery, nitrogen mass balance, and microbial community dynamics. Bioresour Technol. 2018;265:207–13. 10.1016/j.biortech.2018.05.109 29902653

[41] Alegbeleye OO, Singleton I, Sant'Ana AS. Sources and contamination routes of microbial pathogens to fresh produce during field cultivation: A review. Food Microbiol. 2018;73:177–208. 10.1016/j.fm.2018.01.003 29526204 PMC7127387

